# Effects of Whey, Caseinate, or Milk Protein Ingestion on Muscle Protein Synthesis after Exercise

**DOI:** 10.3390/nu8060339

**Published:** 2016-06-03

**Authors:** Atsushi Kanda, Kyosuke Nakayama, Chiaki Sanbongi, Masashi Nagata, Shuji Ikegami, Hiroyuki Itoh

**Affiliations:** Food Science Research Labs., Meiji Co., Ltd., 540 Naruda, Odawara, Kanagawa 250-0862, Japan; kyousuke.nakayama@meiji.com (K.N.); chiaki.sanbongi@meiji.com (C.S.); masashi.nagata@meiji.com (M.N.); shuuji.ikegami@meiji.com (S.I.); hiroyuki.itou@meiji.com (H.I.)

**Keywords:** milk protein, whey protein, caseinate, soy protein, muscle protein synthesis

## Abstract

Whey protein (WP) is characterized as a “fast” protein and caseinate (CA) as a “slow” protein according to their digestion and absorption rates. We hypothesized that co-ingestion of milk proteins (WP and CA) may be effective for prolonging the muscle protein synthesis response compared to either protein alone. We therefore compared the effect of ingesting milk protein (MP) to either WP or CA alone on muscle protein synthesis after exercise in rats. We also compared the effects of these milk-derived proteins to a control, soy protein (SP). Male Sprague-Dawley rats swam for two hours. Immediately after exercise, one of the following four solutions was administered: WP, CA, MP, or SP. Individual rats were euthanized at designated postprandial time points and triceps muscle samples collected for measurement of the protein fractional synthesis rate (FSR). FSR tended to increase in all groups post-ingestion, although the initial peaks of FSR occurred at different times (WP, peak time = 60 min, FSR = 7.76%/day; MP, peak time = 90 min, FSR = 8.34%/day; CA, peak time = 120 min, FSR = 7.85%/day). Milk-derived proteins caused significantly greater increases (*p* < 0.05) in FSR compared with SP at different times (WP, 60 min; MP, 90 and 120 min; CA, 120 min). Although statistical analysis could not be performed, the calculated the area under the curve (AUC) values for FSR following this trend were: MP, 534.61; CA, 498.22; WP, 473.46; and SP, 406.18. We conclude that ingestion of MP, CA or WP causes the initial peak time in muscle protein synthesis to occur at different times (WP, fast; MP, intermediate; CA, slow) and the dairy proteins have a superior effect on muscle protein synthesis after exercise compared with SP.

## 1. Introduction

The turnover of skeletal muscle proteins is regular to the extent that 1%–2% of proteins are synthesized and broken down each day [1]. The turnover of proteins involves ongoing protein synthesis and breakdown, and it is well known that prolonged exercise depresses muscle protein synthesis [2,3].

Previous studies have demonstrated that ingestion of a meal containing protein immediately following exercise stimulates protein synthesis in skeletal muscles [4,5]. More recently, research in mammals has focused on the leucine content of protein due to the central role of this amino acid in muscle protein synthesis in both human and rodent skeletal muscle, by inducing the rapamycin (mTOR) pathway [6,7,8,9,10].

Bovine MP is of the highest nutritional quality because it contains a complete profile of essential amino acids [11]. Milk contains two protein fractions, WP and CA. WP is characterized as a “fast” protein and CA as a “slow” protein because of their digestion and absorption rates [12]. WP contains a higher content of branched-chain amino acids (BCAA), primarily leucine compared to other high quality proteins [13], with its rapid digestion increasing blood amino acid concentrations shortly after ingestion [12,14,15,16]. Several studies have investigated the effect of different protein sources such as whey, soy, caseinate, or wheat on muscle protein synthesis [9,17,18]. These studies demonstrated that WP stimulates muscle protein synthesis to a greater extent than the other protein sources. These results indicated that the greater effect of WP on muscle protein synthesis was due to its high leucine content and rapid aminoacidemia [9,16]. For these reasons, it has been suggested that WP is superior to other protein sources [19,20].

Consumption of WP causes not only hyperaminoacidemia but also additional amino acid oxidation, thereby contributing to a reduction in nitrogen retention [12,14]. On the other hand, ingestion of CA causes slower but prolonged aminoacidemia and it has the best leucine net balance during the postprandial period [12,14]. Ingestion of CA also causes moderate but prolonged muscle protein synthesis compared to WP [21].

It has also been shown that co-ingestion of WP and CA as MP is superior for elevating muscle protein synthesis and muscle accretion after resistance exercise compared to soy protein (SP) [22,23]. Furthermore, CA contributes amino acids that have a prolonged protein-synthetic effect across the leg [24], whereas WP does not.

Taken together, these findings suggest that MP, ~20% WP and ~80% CA, are effective for prolonging muscle protein synthesis response compared to either protein alone.

In addition, some studies demonstrated that a soy-dairy protein blend ingested after exercise was capable of prolonging muscle protein synthesis [25,26]. However, the composition of the soy-dairy protein blend is different from MP. It therefore remains unclear whether MP is superior to WP and CA for stimulating muscle protein synthesis. Therefore, we hypothesized that MP causes a prolonged increase in muscle protein synthesis compared to WP or CA alone. The current study in rats tested this hypothesis by examining whether ingestion of MP increased muscle protein synthesis over a longer period compared to either WP or CA alone after endurance exercise. We also compared the effects of these milk-derived proteins to a SP control.

## 2. Materials and Methods 

### 2.1. Animals

Male Sprague-Dawley rats with a bodyweight of approximately 150 g (CLEA Japan, Inc., Tokyo, Japan, *n* = 237) were used in the study. The rats were maintained at 23 ± 2 °C, with lighting on from 8 a.m. to 8 p.m. and off from 8 p.m. to 8 a.m. The animals had free access to water and food (protein 23.6%, fat 5.3%, carbohydrate 54.4%, ash 6.1%, fiber 2.9%, and moisture 7.7%; MF, Oriental Yeast Co., Ltd., Osaka, Japan). The study was approved by the Animal Committee of the Food Science Research Lab., Meiji Co., Ltd., with the animals receiving care according to the guidelines of this committee (Protocol No. 2012_3871_0174 and 0175, 2013_3871_0028, 0029, 0030, and 0031).

### 2.2. Experimental Protocol 1: Comparison of Different Protein Sources

The swimming exercise protocol was a modification from that used in our previous study [27]. The rats were acclimated to swimming exercise by 30 min of pre-training two days before the experiment. One day before the experiment, the rats were fasted overnight. On the day of the experiment, the rats swam for two hours, with four rats swimming simultaneously in a barrel filled to a depth of 50 cm, providing an average surface area of 400 cm^2^ for each animal. The water temperature was maintained at a constant 35 °C during the swimming protocol. Immediately following exercise, one group of rats was killed (After-Ex, *n* = 8), while the other groups (*n* = 6~8 per group) were given oral administration of one of the following four test solutions: WP concentrate (Tatua Co-operative Dairy Co., Ltd., Morrinsville, New Zealand), caseinate (Fonterra Co-operative Group, Ltd., Auckland, New Zealand), MP concentrate (Fonterra Co-operative Group, Ltd., Auckland, New Zealand), or SP concentrate (Fuji Oil Co., Ltd., Osaka, Japan). The amino acid composition of each protein is shown in Table 1. The macronutrient composition of each protein is shown in Table 2. Each rat was administered an equal amount of protein (2.4 mL/100 g BW, 3.1 g protein/kg BW). The protein content of these preparations was measured by the Kjeldahl method [28].

Individual rats were euthanized at designated postprandial time points (30, 60, 90, 120, 180, and 240 min; *n* = 6~8) under isoflurane anesthesia, and the triceps muscle excised and stored at −80 °C until further use.

### 2.3. Experimental Protocol 2: Dose-Dependent Effects of MP

As mentioned above, the rats swam for two hours on the day of the experiment. Immediately following exercise, rats were given oral administration of one of the following six MP solutions: 0 (0%), 0.39 (12.5%), 0.77 (25%), 1.54 (50%), 3.09 (100%), or 4.63 g protein/kg BW (150%) (2.4 mL/100 g BW, *n* = 8~10). Individual rats were euthanized under isoflurane anesthesia 60 min after ingestion, and the triceps muscle excised and stored at −80 °C until further use.

### 2.4. Administration of Metabolic Tracer

Fifteen minutes prior to sacrifice, a bolus dose (45 mg/kg BW, 22.5 mg/mL) of ^2^H-labeled phenylalanine ([^2^H_5_]Phe, Cambridge Isotope Laboratories, Inc., Tewksbury, MA, USA) was injected via the tail vein to measure the protein fractional synthesis rate (FSR). Fifteen minutes after the injection, the triceps muscle was excised and frozen rapidly. The elapsed time from injection until freezing was recorded as the actual time for incorporation of the labeled amino acid into protein.

### 2.5. Plasma Measurements

Plasma insulin was measured using a commercial ELISA kit for rat insulin (Mercodia AB, Uppsala, Sweden) and plasma free amino acids by high-performance liquid chromatography, with pre-column 6-aminoquinolyl-*N*-hydroxysuccinimidyl carbamate derivatization [29].

### 2.6. Intramuscular Free Amino Acid Concentrations

The supernatant of perchloric acid extracts of triceps muscle was assayed for intramuscular free amino acids by high-performance liquid chromatography, with pre-column 6-aminoquinolyl-*N*-hydroxysuccinimidyl carbamate derivatization [29].

### 2.7. Measurement of Protein Synthesis

The rate of protein synthesis in individual tissues was determined by measuring the incorporation of injected [^2^H_5_]Phe into muscle proteins. Determination of [^2^H_5_]Phe enrichment in plasma and hydrolyzed muscle protein samples was carried out using a modification of the procedure described by Bark *et al.* [30] using a LC/MS/MS system (TQD, Waters Corporation, Milford, MA, USA) and a 2.1 × 50 mm column with a particle size of 1.7 μm (ACQUITY UPLC BEH C18, Waters Corporation, Milford, CT, USA). The mobile phase A consisted of 0.05% trifluoroacetic acid (TFA) in Milli-Q water, and the mobile phase B, 0.05% TFA in acetonitrile. The initial eluent composition was 100% A, followed by an increase to 40% B for 9.0 min, 80% for 1.0 min, and then reduction to 100% of A for 3.0 min. The total running time was 12.0 min, eluent flow 0.3 mL/min, and column temperature 40 °C. The UV trace was recorded at 215 nm, with the analytes detected using electrospray ionization in the positive mode. Multiple-reaction-monitoring was performed using characteristic fragmentation ions (m/z 166.19 > 120.10) for Phe and (m/z 171.19 > 125.10) for [^2^H_5_]Phe. The parameters for the LC/MS/MS analysis were as follows: capillary voltage, 3000 V; source temperature, 120 °C; desolvation temperature, 400 °C; desolvation gas flow, 849 L/h; cone gas flow, 48 L/h; and cone voltage and collision energy set at 25 V and 15 eV, respectively. The fractional rates of protein synthesis (FSR), defined as the percentage of tissue protein renewed each day, were calculated according to the formula:

FSR = (E_b_ × 100)/(E_a_ × t) where t was the time interval between injection and cooling of the tissue sample, expressed in days, and E_b_ and E_a_ were enrichment of [^2^H_5_]Phe in hydrolyzed tissue protein and muscle free amino acids, respectively.

The area under the curve (AUC) was calculated as the overall FSR for each protein treatment to obtain an estimate of the relative abilities of the proteins to stimulate muscle protein synthesis over time.

### 2.8. Western Blotting

The muscle supernatants were subjected to Western blotting as described previously [31]. Phosphorylation of mTOR at Ser2448 was detected using rabbit anti-phospho-mTOR (Ser2448) (Cell Signaling Technology, Danvers, MA, USA) and expressed as the ratio of total mTOR expression, determined using anti-mTOR (Cell Signaling Technology, Danvers, MA, USA). Phosphorylation of Akt at (Ser473) was detected using rabbit anti-phospho-Akt (Ser473) (Cell Signaling Technology, Danvers, MA, USA) and expressed as a ratio of total Akt expression, determined using anti-Akt (Cell Signaling Technology, Danvers, MA, USA).

### 2.9. Statistical Analysis

The data were expressed as means ± SEM. All the statistical analyses were performed using SPSS for Windows, version 14.0J (SPSS Japan).

The data of the comparison of different protein sources were analyzed using two-way ANOVA, and when significant interactions between treatment and time were found, Tukey’s post-hoc analysis was performed for each time point. Differences between the groups were considered to be statistically significant at *p* < 0.05.

The integral for the AUC for %FSR change from baseline (After-Ex) was calculated using the Trapezoidal rule. The data were divided into five intervals between the six time points (30, 60, 90, 120, 180, and 240 min) with the final AUC calculated as the sum of the estimations of the individual time intervals.

The associations between the variables were analyzed using Pearson’s correlation coefficients.

The data of the dose-dependent effects of MP were analyzed using one-way ANOVA, and when significant differences were found, Tukey’s *post-hoc* analysis was performed. Differences between the groups were considered to be statistically significant at *p* < 0.05.

## 3. Results

### 3.1. Fractional Rates of Protein Synthesis (FSR)

**Figure 1:**
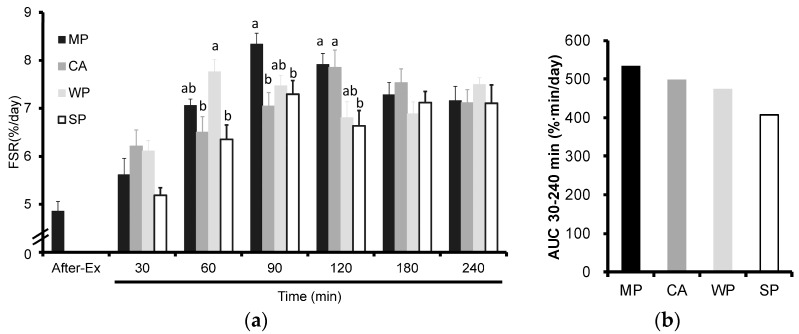
(**a**) Fractional rates of protein synthesis (FSR) in rats administered test solutions with a different protein content. The values are expressed as means (*n* = 6~8), with the standard error shown as vertical bars. Significant time × treatment interactions (*p* < 0.001) were found. ^a, b, c^ Mean values with unlike letters are significantly different (*p* < 0.05; Tukey’s *post-hoc* analysis); (**b**) Area under the curve (AUC) values for the 30–240 min period. MP, milk protein concentrate; CA, caseinate; WP, whey protein concentrate; SP, soy protein concentrate.

As shown in Figure 1a, FSR tended to increase after ingestion of the different types of protein, although the initial FSR peaks were different. MP had a significantly higher FSR compared with either CA or SP at 90 min and with SP at 120 min. WP caused a significant increase in FSR compared with either CA or SP at 60 min. On the other hand, CA caused a significant increase in FSR compared with SP at 120 min. The calculated AUC values for FSR following this trend were: MP, 534.61; CA, 498.22; WP, 473.46; and SP, 406.18 (Figure 1b). No conclusions can be made on these findings because statistical analysis could not be performed. No significant time × treatment interactions were observed in the enrichment of [^2^H_5_]Phe in muscle free amino acids.

### 3.2. Plasma Insulin Levels

**Figure 2:**
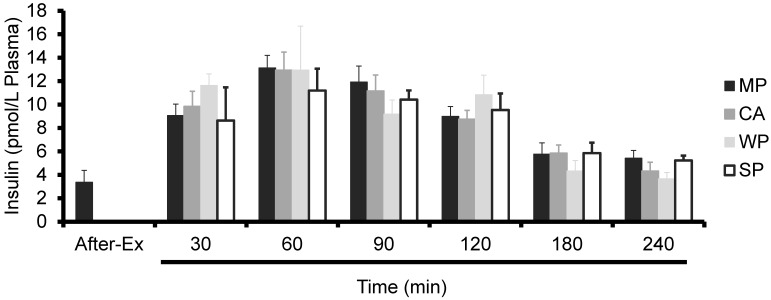
Plasma insulin levels in rats administered test solutions with a different protein content. The values are expressed as mean ± standard error (*n* = 6~8). A significant time effect (*p* < 0.001) but no time × treatment interactions (*p* = 0.90) were observed. MP, milk protein concentrate; CA, caseinate; WP, whey protein concentrate; SP, soy protein concentrate.

Similar to FSR, plasma insulin levels tended to increase in all groups post-ingestion (*i.e.*, a significant time effect; *p* < 0.001). However, no significant time × treatment interactions were observed (Figure 2).

### 3.3. Plasma Amino Acids Levels

**Figure 3:**
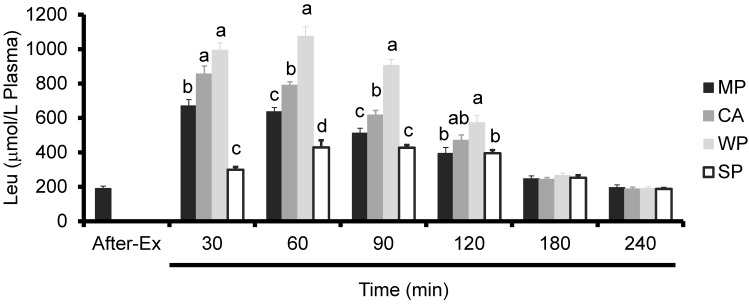
Plasma leucine levels in rats administered test solutions with different protein content. Values are expressed as mean ± standard error (*n* = 6~8). Significant time × treatment interactions (*p* < 0.001) were observed. ^a, b, c^ Mean values with unlike letters are significantly different (*p* < 0.05; Tukey’s *post-hoc* analysis). MP, milk protein concentrate; CA, caseinate; WP, whey protein concentrate; SP, soy protein concentrate.

Plasma leucine levels in all the groups peaked at 60 min. WP produced significantly higher plasma Leu levels than all the other proteins at 60 and 90 min, and compared to MP and SP at 30 and 120 min. CA caused a significant increase in plasma Leu levels compared to MP or SP at 30, 60, and 90 min. On the other hand, MP caused a significant increase in plasma Leu levels compared to SP at 30 and 60 min (Figure 3).

**Figure 4:**
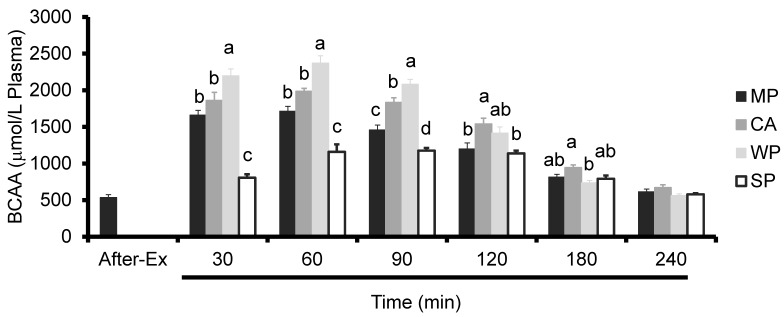
Plasma BCAA levels in rats administered test solutions with different protein content. Values are expressed as mean ± standard error (*n* = 6~8). Significant time × treatment interactions (*p* < 0.001) were observed. ^a, b, c^ Mean values with unlike letters are significantly different (*p* < 0.05; Tukey’s *post-hoc* analysis). BCAA, branched-chain amino acids; MP, MP concentrate; CA, caseinate; WP, whey protein concentrate; SP, soy protein concentrate.

Similar to the trend observed for plasma Leu, the levels of plasma BCAA (isoleucine, leucine and valine) peaked at 60 min in all the groups. WP produced significantly higher plasma BCAA levels compared to all the other groups at 30, 60, and 90 min. CA caused a significant increase in plasma BCAA levels compared to either MP or SP at 90 and 120 min, to SP at 30 and 60 min, and to WP at 180 min. On the other hand, MP caused a significant increase in plasma BCAA levels compared to SP at 30, 60, and 90 min (Figure 4).

### 3.4. Intramuscular Amino Acids Levels

**Figure 5:**
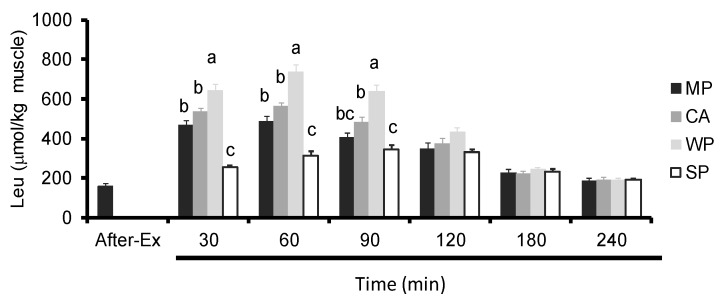
Intramuscular leucine levels in rats administered test solutions with different protein content. Values are expressed as mean ± standard error (*n* = 6~8). Significant time × treatment interactions (*p* < 0.001) were observed. ^a, b, c^ Mean values with unlike letters are significantly different (*p* < 0.05; Tukey’s *post-hoc* analysis). MP, MP concentrate; CA, caseinate; WP, whey protein concentrate; SP, soy protein concentrate.

WP produced significantly higher intramuscular Leu levels compared to all the other groups at 30, 60, and 90 min. CA caused a significant increase in intramuscular Leu levels compared to SP at 30, 60 and 90 min. On the other hand, MP caused a significant increase in intramuscular Leu levels compared to SP at 30 and 60 min (Figure 5).

**Figure 6:**
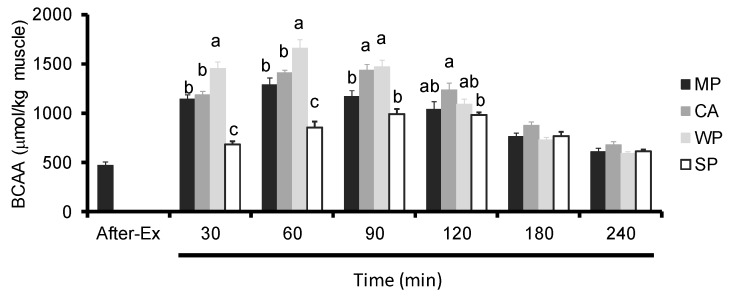
Intramuscular BCAA levels in rats administered test solutions with different protein content. Values are expressed as mean ± standard error (*n* = 6~8). Significant time × treatment interactions (*p* < 0.001) were observed. ^a, b, c^ Mean values with unlike letters are significantly different (*p* < 0.05; Tukey’s *post-hoc* analysis). BCAA, branched-chain amino acids; MP, MP concentrate; CA, caseinate; WP, whey protein concentrate; SP, soy protein concentrate.

Ingestion of WP led to significantly higher intramuscular BCAA levels compared to all other groups at 30 and 60 min, and to MP and SP at 90 min. CA caused a significant increase in intramuscular BCAA levels compared to either MP or SP at 90 min, and to SP at 30, 60, and 120 min. On the other hand, MP caused a significant increase in intramuscular BCAA levels compared to SP at 30 and 60 min (Figure 6).

### 3.5. Dose-Dependent Effect of MP

**Figure 7:**
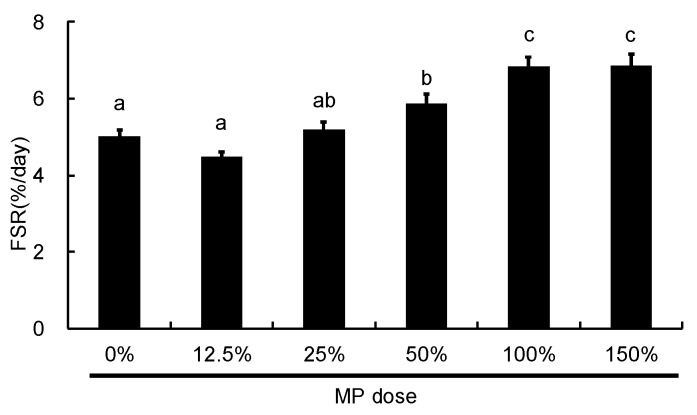
Fractional rates of protein synthesis (FSR) following oral MP administration in rats. FSR was measured 60 min following administration of MP at doses ranging between 0 to 4.63 g protein/kg BW (100% = 3.09 g protein/kg BW). The values are expressed as means (*n* = 8~10), with the standard error shown as vertical bars. ^a, b, c^ Mean values with unlike letters are significantly different (*p* < 0.05; Tukey’s *post-hoc* analysis).

Next, we analyzed the dose-dependent effect of MP on muscle protein synthesis. We found that MP doses between 0% and 100% caused an increase in FSR in a dose-dependent manner, whereas there was no significant difference between the 100% and 150% doses (Figure 7).

**Figure 8:**
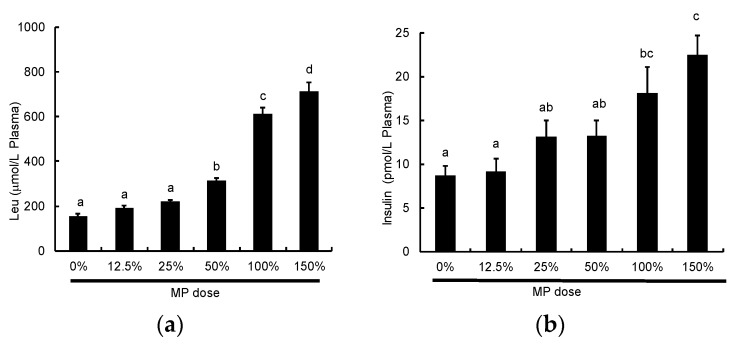
Changes in (**a**) plasma Leu and (**b**) insulin following oral MP administration in rats. Plasma Leu and insulin were measured 60 min following administration of MP at doses ranging between 0 to 4.63 g protein/kg BW (100% = 3.09 g protein/kg BW). The values are expressed as means (*n* = 8~10), with the standard error shown as vertical bars. ^a, b, c, d^ Mean values with unlike letters are significantly different (*p* < 0.05; Tukey’s *post-hoc* analysis).

We also analyzed the dose-dependent effect of MP on plasma Leu and insulin levels. MP caused an increase in both plasma Leu and insulin levels in a dose-dependent manner (Figure 8a,b).

**Figure 9:**
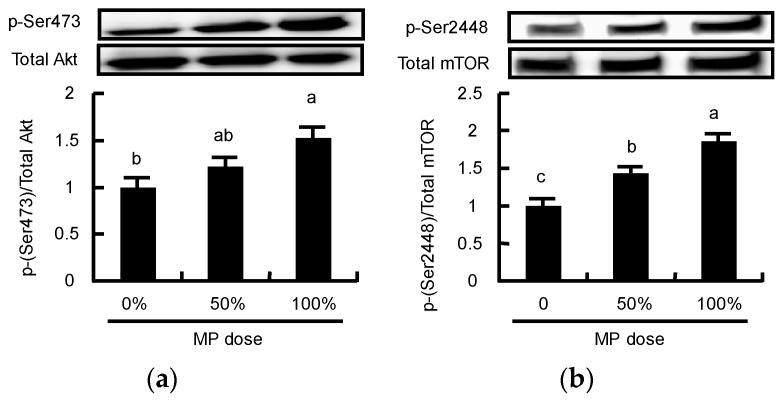
Changes in (**a**) the phosphorylation state of Akt and (**b**) mTOR following oral MP administration in rats. Phosphorylation of Akt and mTOR was measured 60 min following administration of MP at doses ranging between 0 to 4.63 g protein/kg BW (100% = 3.09 g protein/kg BW). The values are expressed as means (*n* = 8~10), with the standard error shown as vertical bars. ^a, b, c,^ Mean values with unlike letters are significantly different (*p* < 0.05; Tukey’s *post-hoc* analysis).

Finally, we analyzed the phosphorylation of Akt and mTOR to determine whether MP activated these proteins involved in mTOR signaling in a dose-dependent manner. MP caused greater phosphorylation of Akt at a dose of 100% compared to a dose of 0% (Figure 9a). MP also caused greater phosphorylation of mTOR in a dose-dependent manner (Figure 9b).

## 4. Discussion

This is the first study to compare the effects of ingestion of MP to either WP or CA alone on muscle protein synthesis after exercise. As reported in other studies, we showed milk-derived proteins (MP, CA, and WP) caused a greater increase in FSR compared to SP [18,22,23]. We also demonstrated that ingestion of MP, CA, or WP resulted in different times for initial peak muscle protein synthesis to occur (WP, 60 min; MP, 90 min; CA, 120 min) and that ingestion of MP caused a prolonged increase in muscle protein synthesis compared to SP at 90 and 120 min. On the other hand, WP or CA alone caused a short-term increase in FSR compared to SP (60 min for WP and 120 min for CA). In addition, the calculated AUC values for FSR following this trend were MP, 534.61; CA, 498.22; WP, 473.46; and SP, 406.18, although no conclusions can be made on these findings. These results indicated that the dairy proteins have a superior effect on muscle protein synthesis after exercise compared with SP. Furthermore, it can be possible that MP (a combination of WP and CA) is an ideal protein source for muscle protein synthesis following exercise compared to either WP or CA alone. Further studies are required to compare particularly the effects of these dairy proteins on FSR.

It is well known that WP is characterized as a “fast” protein and CA as a “slow” protein because of their digestion and absorption rates [12]. There is also considerable evidence that ingestion of WP causes a rapid and transient increase in muscle protein synthesis [9,21]. In this study, we also showed that WP caused a greater increase in FSR compared to SP at 60 min, although there was no significant difference in FSR between the two proteins after 90 min. On the other hand, CA showed that the peak FSR was delayed until 90 min, with this increase being greater than that observed for SP at the same time. These results are consistent with those reported by a previous study by Reitelseder *et al.* [21] who showed that the peak muscle FSR in human subjects was delayed after consumption of CA compared to WP. Furthermore, in that study, CA caused a prolonged increase in FSR compared to controls for up to 6 h. On the other hand, WP caused an increase in FSR compared to the control for only 3.5 h. We also showed that MP, a blend of CA and WP, caused a prolonged increase in FSR compared to SP. Reidy *et al.* [26] reported that protein blends caused a prolonged FSR response compared to a single protein source. However, that study used a soy-dairy protein blend that had a different protein composition (WP:CA:SP of 25:50:25) from MP. Further studies are therefore needed to compare the effects of a dairy protein blend (MP) to a soy-dairy protein blend and identify the ideal protein source for muscle protein synthesis.

Although there were no significant differences between the protein groups, ingestion of MP, CA, or WP caused differences in the response time of plasma insulin levels. WP induced a rapid increase in insulin levels at 30 min compared to SP, although this increase was transient. On the other hand, MP and CA caused a slow but prolonged increase in plasma insulin levels between 60–90 min compared to SP. It appeared that these changes in plasma insulin levels occurred before the changes in FSR. Previous studies have indicated that a minimal concentration of plasma insulin is required for both amino acid- and exercise-induced stimulation of protein synthesis in skeletal muscle [32]. It is also well known that MPs have insulinotropic properties, with WP being a more efficient insulin secretagogue than other protein sources [29,33,34]. Taken together, it is possible that different changes in plasma insulin levels caused by MP, CA, or WP may have different effects on muscle protein synthesis. However, we showed no significant difference in plasma insulin levels in any of the groups in our study. Further studies are therefore required to compare the effects of MP, CA and WP on insulin secretion.

It is well known that BCAA, especially leucine, plays an important role in the activation of muscle protein synthesis [35,36]. Leucine alone or as a supplement has been shown to stimulate muscle protein synthesis after exercise in models of either endurance or resistance exercise [37,38]. This led Norton *et al.* to suggest that peak activation of muscle protein synthesis was proportional to the leucine content of a meal [9]. In the current study, the plasma leucine levels in each of the protein groups corresponded relatively closely to the leucine content of the different proteins, such that plasma leucine levels were highest for WP, intermediate for MP and CA, and lowest for SP. However, we showed no positive correlation between FSR and either plasma (*p* = 0.53) or intramuscular leucine levels (*p* = 0.18). In the Experiment 2, we examined different doses of MP and found that mTOR activation reaches a plateau at 100% MP. The doses of 50%, 100% and 150% MP contained ~22 mg, 43 mg and 65 mg Leu, respectively. While these doses are relatively far apart, the data suggest that there is a leucine threshold around 43 mg with a detectable difference at 22 mg. In Experiment 1, the four treatments were MP, CA, WP and SP, and the leucine amounts were ~43 mg, 41 mg, 55 mg and 36 mg, respectively. With no threshold titration study or initiation factor data (S6K1 or 4E-BP1), we assume that MP, CA, and WP are all activating the mTOR signaling, while the 36 mg of SP is below full activation. Thus, while there are differences in plasma and intramuscular leucine concentrations across the treatments, the differences are irrelevant because mTOR is fully activated by the 43 mg dose. These results are consistent with previous studies that indicated there was a threshold level of leucine required to stimulate mTOR signaling, and once this level was achieved, further increases in leucine did not result in an increase in the muscle anabolic response [9,39,40,41,42]. Further studies are therefore required to compare the effects of MP, WP, or CA at lower doses than those used in the current study.

The measure limitation of this study is that we failed to measure mTOR or associated initiation factors in the central experiment (Experiment 1). mTOR signaling is a key regulatory factor for muscle protein synthesis and is regulated positively by amino acids, particularly leucine [43]. In the present study, ingestion of MP caused a greater increase in plasma Leu levels and greater phosphorylation of mTOR levels in a dose-dependent manner. However, it remains unclear whether ingestion of MP, CA or WP causes the initial peak time in the phosphorylation of mTOR levels to occur at different times (WP, fast; MP, intermediate; CA, slow). Further studies are needed to demonstrate the effect of MP on mTOR signaling compared with either WP or CA alone.

## 5. Conclusions 

This is the first study to compare the effect of ingestion of MP to either WP or CA alone on muscle protein synthesis after exercise. We demonstrated that the ingestion of MP, CA or WP causes the initial peak time in muscle protein synthesis to occur at different times (WP, fast; MP, intermediate; CA, slow). We also demonstrated that the dairy proteins have a superior effect on muscle protein synthesis after exercise compared with SP. The findings of the present study provide new insights into the effects of different protein sources for use in sports nutrition.

## Figures and Tables

**Table 1:** Amino acid composition of the test proteins.

	MP	CA	WP	SP
	g/100 g			
Ala	3.14	2.81	4.69	4.15
Arg	3.30	3.50	2.73	7.58
Asx	7.33	6.71	11.22	11.66
Cys	0.70	0.32	2.99	1.25
Glx	20.62	21.25	16.27	19.50
Gly	1.77	1.71	1.97	4.12
His	2.81	2.89	2.31	2.63
Ile	4.92	4.86	5.31	4.55
Leu	9.34	8.81	11.89	7.84
Lys	7.75	7.57	9.39	6.20
Met	2.58	2.72	2.15	1.27
Phe	4.66	4.82	3.61	5.23
Pro	9.48	10.13	4.60	5.27
Ser	5.19	5.26	4.52	5.09
Thr	4.20	4.09	5.19	3.84
Trp	1.29	1.14	2.27	1.36
Tyr	4.84	5.22	3.61	3.74
Val	6.08	6.19	5.27	4.72

MP, milk protein concentrate; CA, caseinate; WP, whey protein concentrate; SP, soy protein concentrate.

**Table 2:** Macronutrient profile of the test proteins.

	Carbohydrate	Protein	Fat	Energy
	(g/100 g)			(kcal/100 g)
MP	9.2	76.7	1.4	356.2
CA	0.1	91.4	0.7	372.3
WP	6.6	79.3	5.9	396.7
SP	4.0	85.8	0.2	361.0

MP, milk protein concentrate; CA, caseinate; WP, whey protein concentrate; SP, soy protein concentrate.

## Abbreviations

The following abbreviations are used in this manuscript:

BCAA	branched-chain amino acids
CA	caseinate
MP	milk protein
mTOR	mammalian target of rapamycin
WP	whey protein
